# TEAMS (Tele-Exercise and Multiple Sclerosis), a Tailored Telerehabilitation mHealth App: Participant-Centered Development and Usability Study

**DOI:** 10.2196/10181

**Published:** 2018-05-24

**Authors:** Mohanraj Thirumalai, James H Rimmer, George Johnson, Jereme Wilroy, Hui-Ju Young, Tapan Mehta, Byron Lai

**Affiliations:** ^1^ Department of Health Services Administration University of Alabama at Birmingham Birmingham, AL United States; ^2^ UAB/Lakeshore Research Collaborative School of Health Professions University of Alabama at Birmingham Birmingham, AL United States

**Keywords:** multiple sclerosis, exercise, therapy, mHealth, user-centered design

## Abstract

**Background:**

People with multiple sclerosis face varying levels of disability and symptoms, thus requiring highly trained therapists and/or exercise trainers to design personalized exercise programs. However, for people living in geographically isolated communities, access to such trained professionals can be challenging due to a number of barriers associated with cost, access to transportation, and travel distance. Generic mobile health exercise apps often fall short of what people with multiple sclerosis need to become physically active (ie, exercise content that has been adapted to accommodate a wide range of functional limitations).

**Objective:**

This usability study describes the development process of the TEAMS (Tele-Exercise and Multiple Sclerosis) app, which is being used by people with multiple sclerosis in a large randomized controlled trial to engage in home-based telerehabilitation.

**Methods:**

Twenty-one participants with disabilities (10 people with multiple sclerosis) were involved in the double iterative design, which included the simultaneous development of the app features and exercise content (exercise videos and articles). Framed within a user-centered design approach, the development process included 2 stages: ground-level creation (focus group followed by early stage evaluations and developments), and proof of concept through 2 usability tests. Usability (effectiveness, usefulness, and satisfaction) was evaluated using a mixed-methods approach.

**Results:**

During testing of the app’s effectiveness, the second usability test resulted in an average of 1 problem per participant, a decrease of 53% compared to the initial usability test. Five themes were constructed from the qualitative data that related to app usefulness and satisfaction, namely: high perceived confidence for app usability, positive perceptions of exercise videos, viable exercise option at home, orientation and familiarity required for successful participation, and app issues. Participants acknowledged that the final app was ready to be delivered to the public after minor revisions. After including these revisions, the project team released the final app that is being used in the randomized controlled trial.

**Conclusions:**

A multi-level user-centered development process resulted in the development of an inclusive exercise program for people with multiple sclerosis operated through an easy-to-use app. The promotion of exercise through self-regulated mHealth programs requires a stakeholder-driven approach to app development. This ensures that app and content match the preferences and functional abilities of the end user (ie, people with varying levels of multiple sclerosis).

## Introduction

Regular participation in physical activity continues to gain recognition as a behavioral approach that can safely improve, or help alleviate, both functional (eg, reduced balance and walking capacity) and symptomatic consequences (eg, severe fatigue, depression, and cognitive dysfunction) of multiple sclerosis (MS) [1-3]. However, adults with MS are less physically active than adults without disabilities, with only 20% of adults with MS meeting the US national guidelines of moderate-to-vigorous physical activity required to improve and maintain health [4,5]. Low physical activity participation can lead to the onset or exacerbation of common secondary conditions experienced by people with MS [6], which include pain, fatigue, deconditioning, weakness, falls, and depression [6-9]. One or more of these health conditions can have a negative impact on health and function across the lifespan and, in the aggregate, can limit or restrict participation in general life activities including employment, social and community engagement, and performing instrumental activities of daily living [10-13].

People with MS face varying levels of disability and symptoms, and thus require highly trained therapists and/or exercise trainers specialized in disability populations (eg, Certified Inclusive Fitness Trainers through the American College of Sports Medicine or Certified Special Population Specialists through the National Strength and Conditioning Association) to design personalized exercise programs. Unfortunately for people living with MS in geographically isolated communities, access to trained professionals is limited or nonexistent. This has increased the need for better delivery methods to reach people with MS who do not have access to appropriate care.

Several studies have reported that delivering home-based therapeutic exercises through information and communication technology, referred to as telerehabilitation, can be an equally effective alternative to onsite rehabilitation in the delivery of care to hard-to-reach populations [14-17]**.** The incorporation of this technology in addressing the needs of people with MS has the potential to greatly improve their access to exercise and rehabilitation, while eliminating the barriers of time, cost, and personnel (ie, driver or caregiver) associated with onsite rehabilitation [18].

Mobile health (mHealth) apps can provide a ubiquitous channel for delivering convenient and personalized telerehabilitation to people with MS [19]. These apps can enable researchers to deliver and quantify “precise” doses of exercise that are customized to the unique needs of the end user and can be accessed in the comfort of one’s home [20]. Researchers can also increase the likelihood that participants will engage in the exercise behavior offered by the app by embedding behavior change theory principles within the mHealth app.

One of the more popular theories of behavior change is the Social Cognitive Theory (SCT) [21]. Components of SCT include self-regulation or monitoring, goal-setting, informational advice, and role-modeling [22,23]. While embedding SCT into new mHealth apps increases the potential viability of the product over the long term, in the short term promoting exercise behavior ultimately depends on participants’ perceptions of how easy it is to use the app [24].

While there are thousands of commercially available exercise apps for the general population, there are few, if any, that have been specifically designed for people with MS. Therefore, the purpose of this usability study is to describe the development of a therapeutic exercise app for people with MS. The study is part of an ongoing, multisite randomized controlled trial across 38 locations in Alabama, Mississippi, and Tennessee. The Patient-Centered Outcomes Research Institute (PCORI)-funded study is referred to as the TEAMS project, which stands for Tele-Exercise And Multiple Sclerosis.

## Methods

### Design

The study incorporated a parallel-iterative design, whereby the project team simultaneously developed both the app features and the exercise content (videos and articles).

### User-Centered Design Features

An app that would be useful to people with different functional levels of MS requires a user (participant)-centered design (UCD) that involves their input early in the development process [25]. The choice of a UCD was made to ensure that the app was usable and acceptable to people with MS. Furthermore, the entire project is grounded on patient centeredness. Four UCD principles were employed during this study: (1) early input or feedback from users; (2) tests of usability through quantitative (eg, surveys or questionnaires) and qualitative data (eg, observational or verbal feedback); (3) iterative tests and design; and (4) an integrated design that allows usability issues to be identified and addressed concurrently with the development of the product.

### Project Team

Development of the app included 2 project teams (research and development). The research team included 4 members with backgrounds in exercise and recreational technology for people with disabilities. The development team consisted of 2 app developers led by a Health Informatics researcher. The research team conducted and analyzed the evaluation phases (focus group interviews and usability testing) and worked in parallel with the development team throughout the creation of the TEAMS app. Members of both the research and development team conducted the heuristic analysis. The creation of content included an adapted exercise specialist (5 years of experience) and occupational therapist (21 years of experience), which were informed by a stakeholder group and a clinical consultant group. Stakeholders consisted of 10 individuals with MS, researchers, and therapists. Specifically, 5 of the stakeholders are individuals with MS, 2 are health care providers for individuals with MS, one is a caregiver for a person with MS and 2 represent national and regional MS specific organizations. The project consultant group included 2 clinicians, 5 data safety monitoring board members, and a study consultant who was a senior researcher in exercise science for people with MS and contributed to the development of exercise content. The stakeholder group was created specifically for this project to oversee the creation of the app and content, as well as the implementation of the project. Stakeholders were heavily involved with the development of the app content and oversaw the development of the app through monthly meetings or workshops, which members of each project team attended.

### Instruments

**Figure 1:**
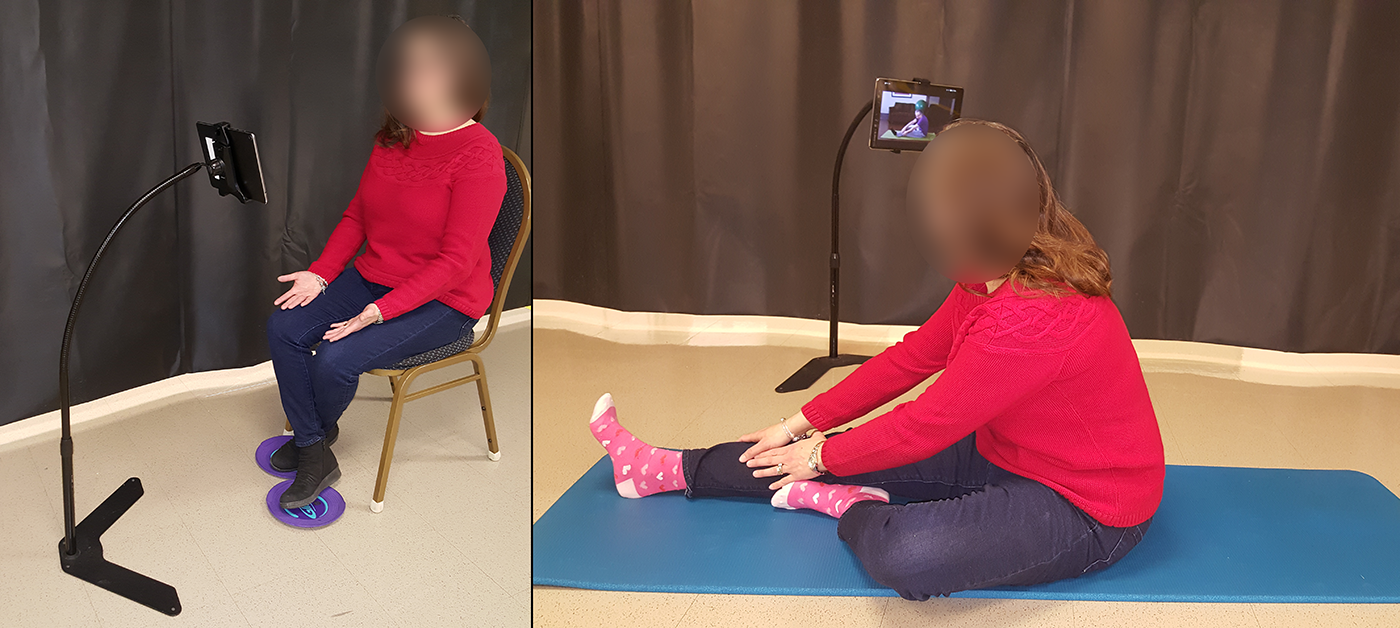
Tablet and table stand setup used to complete exercises in the seated position and on the floor.

The project aim was to create a comprehensive home tele-exercise program that could be performed by individuals with varying levels of MS using an Android computer tablet (Asus ZenPad 3s 10) and an adjustable floor stand (Standzout Standzfree 48” Universal Pro Tablet Floor Stand). The tablet floor stand could be adjusted to accommodate various exercise positions (lying on the floor, seated, and standing). An example of the setup is shown in Figure 1. Specifically, the custom-designed Android app provided an easy-to-use interface for navigating and viewing the exercise videos. The app is built to operate on any Android or iOS device (of any size) that is a phone or tablet. The app is, and will be, available free of charge. The Android version is currently available for public download; however, users will not be able to sign up for an account until the clinical trial is completed.

### TEAMS Development Process

**Figure 2:**
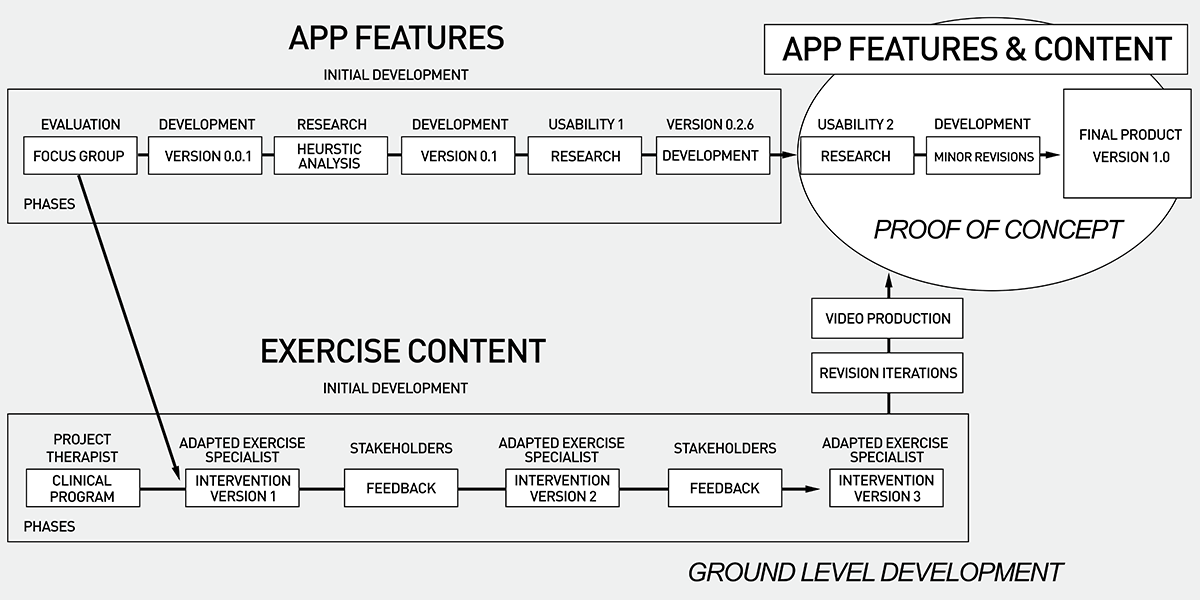
An overview of the TEAMS development process.

An overview of the entire development process is displayed in Figure 2, which occurred between the years 2016 to 2017 and involved two phases: (1) ground-level creation of app features and exercise content, and (2) proof of concept trials through individual user testing.

#### Participants

A range of people with disabilities, including people with MS, were recruited for the study to ensure that multiple functional levels were addressed in the design of the app. Twenty-one participants participated in 1 focus group and 2 individual usability tests. Sample sizes for the usability tests were based on saturation of the qualitative data [26]. This criterion involved completing the usability tests until there were no new themes identified.

Eligibility criteria included: (1) minimum age of 18 years, (2) residence in the local metro area, (3) documented mobility limitation, (4) ability to speak and understand English, and (5) ability to operate an app on a mobile device. In addition, both the focus group and the individual user tests required at least one individual who used a wheelchair. None of the participants had any experience with the mobile app evaluated in the study. The Institutional Review Board of the University of Alabama at Birmingham approved the protocol. Prior to enrollment, written consent was obtained from each participant.

#### Ground-Level Creation of App Features and Exercise Content

The exercise content team created exercise videos and articles that could be delivered periodically throughout a 12-week program. The exercise videos included yoga, Pilates, and dual-task exercises, which were derived from an onsite program that was developed in 2010 by a group of certified MS Clinical Specialists at a comprehensive MS care center. The exercise content was further adapted to target 4 functional levels of people with MS based on stakeholder feedback and literature review. Every exercise was designed to also include a challenged version and a modified version by using exercise equipment to increase exercise intensity or assist with movement. The exercise equipment included yoga mat, a Swiss ball, half roll, sliders, a small inflatable ball, yoga straps, yoga block, a Pilates disc, and resistance bands. Articles and infographics were also included in the app focusing on exercise, and self-regulatory strategies such as goal-setting, seeking social support, and overcoming barriers. The articles were modified from publicly available information on the National Center on Health, Physical Activity and Disability website (www.nchpad.org) [27].

After embedding the exercise videos and articles into the app, stakeholders and study consultants viewed and tested the content and provided feedback and suggestions for further adaptations. Stakeholder feedback resulted from formal monthly meetings and informal individual meetings that included pilot testing. The adapted exercise specialist recorded stakeholder feedback through written qualitative and observational notes.

##### Focus Group

The development team began ground-level creation of the TEAMS app from the feedback received from the focus group interview. The purpose of the focus group was to identify what features needed to be included in the initial version of the app. The focus group was held at a community fitness facility. A member of the research team guided the group and took written notes. The group interview contained 6 open-ended questions, which served as general prompts for discussion in the following areas: general types of exercise performed; challenges or issues experienced towards exercise participation; perceptions, experiences, and preferences of using fitness apps; challenges or issues experienced with fitness apps; and preferences for app features. Participant responses were probed in further detail through several follow-up questions. An audio recording device was used to capture participant feedback, which was later transcribed for analysis. The development team utilized the focus group results to build the initial features and user interface of the TEAMS app version 0.1.

##### Heuristic Analysis

The development team conducted a rapid heuristic analysis to assess general usability issues in the app version 0.1. The heuristic evaluation involved the collaborative efforts of both the development and research teams evaluating the app against accepted usability principles [28]. Any deviations from these accepted principles were referred to as a heuristic violation. Each heuristic violation was assigned a severity rating from 1 to 4, with 4 being the most severe. This heuristic analysis was performed in accordance with Nielsen's 10 Usability Heuristics for User Interface Design [29]. Following the heuristic analysis, the development team further refined the app version 0.1.

After initial versions of the app content and features were created, they were combined for usability testing in individuals with MS. Data recorded from usability tests included both quantitative and qualitative data (ie, a pragmatic mixed methods approach, namely “quan+QUAL”), which were selected or mixed at each evaluation phase to expand our understanding of app usability [30]. The project team anticipated that the creation of the TEAMS app and content would require several iterations and continued the research using the aforementioned UCD principles [31] and saturation point for data completion. This process resulted in a total of 2 usability tests and 2 further development iterations to the app features. Based upon user feedback, new or advanced adaptations to the original exercise content were not warranted.

##### Usability Test One

Following app revisions, newly recruited participants completed individual usability testing of the latest app (version 0.2). A concurrent thinking aloud process was used, whereby participants were asked to complete representative tasks on an Android tablet, while a researcher observed their actions and asked questions [32]. Participants performed 8 usability tasks, which involved navigating various app pages and testing app functions. Participants were also instructed to locate, play, and perform an exercise video that they felt was suitable to their functional ability. As a preliminary test of app function, exercise content was not included within the first usability test. A research assistant took written notes from their observations of participants during the usability tasks, which were later qualitatively analyzed. The research assistant also recorded the frequency, nature, and location of issues that participants encountered during the usability tasks. These issues included those explicitly reported by participants, as well as the issues observed by the research assistant.

###### Usability Test Two

After the development team made substantial revisions to the app (version 0.2.6), the research team conducted a second round of usability testing. The second usability test protocol matched that of the former, except for additional tasks to accommodate new app functions and a post-data collection qualitative interview. The interview was added because the development team determined that the app was a near-finalized product based upon results from the first usability test. The face-to-face interview was conducted in a comfortable setting that was chosen by the participant. The goal of this interview was to understand participants’ perceptions of the app and determine whether further development changes to the app were necessary. The interview was recorded by an audio device, which was later transcribed for qualitative analysis.

#### Proof of Concept

##### Usability Measures

App usability can be defined in terms of effectiveness (ie, the ease at which individuals can use the product in a manner they expect), usefulness (ie, the extent a product can enable users to achieve their goals and willingness to use the product), and satisfaction (ie, the users’ perceptions and opinions of the product) [31].

Accordingly, this study assessed three core areas of usability: *effectiveness*, *usefulness*, and *satisfaction.* Researchers evaluated *effectiveness* by observing and recording the frequency of problems that participants experienced during the usability tasks [31]. Since a different sample size was recruited for the usability tests, the research team recorded the mean number of identified problems per participant. Each problem that participants identified was classified under the Usability Problem Taxonomy [33]. This taxonomy allowed usability problems to be classified into both a task and artifact component. The artifact component included problems with visualness, language, and manipulation of the product. In contrast, the task component involved problems related to task-mapping (ie, interaction, navigation, or function) and task-facilitation (eg, task automation, user action reversal, and keeping the user on task). The Usability Problem Taxonomy was incorporated to help the development team pinpoint direct versus implied fixes.

Researchers assessed *usefulness* and *satisfaction* through participants’ perceptions of completing the usability tasks via a face-to-face interview. Accordingly, the interview included questions that sought to gain insight into participants’ overall perceptions of the app, including its usefulness, their likes and dislikes regarding app features and usability, their suggestions for improvements to the app, and their experience performing exercise videos. Additionally, participants were also asked whether they thought the app was ready to be delivered to the public. This was used as an indicator of whether further revisions to the app or usability tests were necessary. Two members of the research team conducted the interviews. One interviewer was a research staff member that was trained and supervised by the primary interviewer. The primary interviewer had 3 years of experience with qualitative interviews and had a background in adapted physical activity.

##### Usability Setup and App Content

Equipment included a 10.5-inch Android tablet that was mounted on an adjustable floor stand (Standzfree Universal Stand, Standzout) and came installed with the TEAMS app. The app included the exercise videos and articles that resulted from the ground-level development stage. Specifically, the app included 2 articles and 6 sample playlists of exercise videos, one for each functional level that resulted from the exercise content development process. The app also featured a home page with weekly instructions and goals, earnable badges that were awarded for completing specific tasks such as reading an article, a built-in calendar, and notifications that informed users of newly added content. If required in the video, participants used the following exercise equipment: a Swiss ball, yoga mat, sliders, a small inflatable ball, and resistance bands.

#### Analysis

The research team’s philosophical assumptions aligned with dialectical pluralism [34]. Dialectical pluralism provides a way for researchers, practitioners, clients, policy makers, and other stakeholders to work together and produce new workable “wholes” while concurrently thriving on differences and intellectual tensions. Within this paradigm, the research team held separate theoretical perspectives when analyzing the quantitative and qualitative data. Quantitative data were descriptively reported. Researchers also recorded the age, disability or condition, and mobility information from all participants involved throughout the research process (ie, people who participated in the focus group and usability tests).

Qualitative data were analyzed using inductive thematic analysis [35] framed within Interpretivism (ontological relativism and epistemological subjectivism) [36]. Within this process, the research team first transcribed the qualitative data and then checked the transcriptions for accuracy with the audio recordings. Next, two analysts generated initial codes from segments of transcribed interviews or written notes. These codes were then refined into fewer subthemes for a single transcription. The analysts repeated this process for each transcription and evolved their themes in a case-by-case manner. The analysts then met to discuss their subthemes, which were then integrated and refined into a single set of major themes based on internal and external homogeneity [37]. During this process the analysts acted as “critical friends” [38]. Thus, they voiced their interpretations of the data and underwent critical discussions based upon their epistemological beliefs, with the goal of reaching the most plausible interpretation of the data. One analyst, the primary interviewer, had 5 years of clinical experience in exercise training for people with disabilities and had a background in mixed-methods research. The other analyst also had a background in mixed-methods research, which focused on the development of a grounded theory model to inform adaptive intervention designs for increasing physical activity in people with disabilities.

## Results

### Overview

Participant demographics for the focus group and usability tests are shown in Table 1, and their clinical characteristics are shown in Table 2. Overall, 21 people with disabilities were involved in the entire research process (mean age 54 years, SD 2; 14 females, 8 males). The 8 individuals included in the focus group represented several functional levels of disability. Eight people with disabilities were included in the first usability test. The final usability test included 5 people with MS who had varying levels of functional mobility.

The following subsections include summaries of the results and accompanied revisions or changes that were made by the project team for both the app features and content.

### App Features Findings

#### Focus Group

Focus group participants noted the following qualitative themes: *barriers to exercise, disability-specific exercise content,* and *suggestions for app features*. The *barriers to exercise* theme included several issues with exercise onsite at a fitness facility such as lack of time, convenience, and transportation. To circumvent these issues, participants identified that the home environment was an ideal setting for exercise. However, participants noted that they required *disability-specific exercise content*, which they currently had limited access to. One participant stated:

You can find stuff on the internet but some of it you can't adapt [for people with disabilities].

Specifically, they desired exercises that were suitable to their functional needs and could lead to health benefits or improvements in fitness.

**Table 1:** Demographics of participants included in the focus group and usability tests.

Participant demographics		Focus group (N=8)		Usability test 1 (N=8)		Usability test 2 (N=5)	
**Sex, n (%)**							
	Male		2 (25)		4 (50)		1 (20)
	Female		6 (75)		4 (50)		4 (80)
Age (years), mean (SD)		54 (11)		54 (13)		53.6 (8.5)	
**Ethnicity, n (%)**							
	Non-Hispanic white		6 (75)		5 (62.5)		2 (40)
	Black		2 (25)		3 (37.5)		3 (60)

**Table 2:** Clinical characteristics of participants the focus groups and usability tests.

Clinical characteristics		Focus group (N=8)	Usability test 1 (N=8)	Usability test 2 (N=5)
**Disability, n (%)**				
	Spinal cord injury	2 (25)	1 (12.5)	N/A^a^
	Multiple sclerosis	2 (25)	3 (37.5)	5 (100)
	Cerebral palsy	1 (12.5)	2 (25)	N/A
	Parkinson disease	1 (12.5)	N/A	N/A
	Spina bifida	1 (12.5)	N/A	N/A
	Vision impairment	1 (12.5)	N/A	N/A
	Stroke	N/A	1 (12.5)	N/A
	Hypertension	N/A	1 (12.5)	N/A
**Mobility device, n (%)**				
	Cane	4 (50)	2 (25)	1 (20)
	Power wheelchair	1 (12.5)	2 (25)	1 (20)
	Manual wheelchair	2 (25)	1 (12.5)	N/A
	Orthotic device	1 (12.5)	N/A	N/A
	Independent ambulator	N/A	2 (25)	1 (20)
	Walker	N/A	1 (12.5)	2 (40)

^a^N/A: not applicable.

**Table 3:** The Usability Problem Taxonomy (UPT) results from the usability tests.

Characteristics		Usability test 1 (n=8)	Usability test 2 (n=5)
Problems per user, mean (range)		2.13 (1-6)	1.0 (1-2)
Severity, mean (range)		2.17 (1-4)	1.5 (1-2)
**Location or screen (area=# of problems)**			
	Calendar	3	4
	Articles	2	1
	Menu	6	–
	All Screens	4	–
	Videos	2	–
	Profile	1	–
**UPT Artifact Classification (type=# of problems)**			
	Visualness	5	3
	Manipulation	5	2
	Language	7	–
**UPT Task Classification**			
	Mapping	12	2
	Facilitation	5	3

Participants also desired exercises that could be performed with limited equipment but were also challenging and, most importantly, enjoyable.

Regarding *suggestions for app features*, participants expressed the desire to be able to enter their personal level of physical function into an app and receive a customized workout program based on the type of activities they could perform. They also acknowledged that observing other people with similar functional abilities perform exercises enhanced their motivation to exercise. Additionally, participants stated a desire for app features that could enhance motivation to exercise through easily obtainable achievement rewards. Based upon these themes, the app development team oriented the initial development of the TEAMS app to include the following features: an app that could be instantly tailored to the functional needs of an individual (ie, an app that could quickly alternate between exercise programs of various challenges or movement adaptations), downloadable exercise videos that were directed or modeled by people with MS, and earnable badges for completion of achievements.

#### Heuristic Analysis

The heuristic analysis resulted in 18 violations with an average *moderate* severity of 2.5 [29,39,40]. The most commonly violated heuristic item was the *match between system and the real world*. In other words, it was suggested that the language used in the app should be changed to reflect everyday user language, instead of system-oriented terms. The most severe violations included the absence a help menu or tutorial, the inability to open a social media post from another user, and the inability to confirm the entry of social media posts. These finding were presented to the development team, which were subsequently integrated into the next version of the app.

#### Usability Test One

Results from usability tests 1 and 2 are shown in Table 3. The first round of usability testing resulted in an average of 2.13 problems (range 1-6) per participant (15 problems identified by 8 people), with an average severity score of 2.17. Users identified several problems throughout different locations of the app, with most problems located in the Main Menu and Calendar. These issues were related visualization, language, and manipulation, which primarily interfered with task mapping (ie, navigation, function, and interaction). Based upon these results, the development team underwent a complete overhaul of the aesthetics (color, font size, the addition or revision of icons) and problem areas. For example, the default text size within the tablet was increased to the largest size setting, and the Main Menu included weekly instructional content and notifications for newly added video and article content. The development team also made efforts to enhance verbal cues and task navigation by the addition of icon images and more noticeable fonts for buttons.

#### Usability Test Two

The second round of usability testing resulted in an average of 1.0 problems per participant and were of low severity. Most problems were located in the Calendar menu and were related to visualization, which caused issues both with task mapping and facilitation (eg, staying on task). These results informed the development team that the calendar still required changes to enhance the user-experience, which were included in the app version 1.0.

Qualitative analysis resulted in 6 themes: *high-perceived confidence for app usability, positive perceptions of exercise videos*, *viable exercise option for the home, orientation and familiarity required for successful participation, app issues,* and that the app was *ready to be delivered to the public after minor revisions.* Specifically, all participants felt confident that they could operate the app independently and noted that the app was similar to other Android apps in the marketplace.

Regarding the exercise content, participants appreciated the precision of the exercise videos that allowed for a wide variety of individuals with different functional abilities to perform the exercise routines. Participants also acknowledged potential benefits of the exercises and that the movements were similar to their past experiences in therapy:

I would love to do this because I know it helps your balance… I think the exercise videos are good, because a lot of the movements are what you do in therapy. So, this is along that line to get you moving more

Participant 4

Due to these positive perceptions, participants stated that the app was a viable exercise option for individuals with MS to perform at home. Interestingly, participants noted that the app should act as a supplement or transition towards engagement in community exercise (ie, not a substitute). Community engagement fosters interpersonal relationships with the instructor and other members or participants.

Participants identified several minor *app issues*. First, engagement in the program would require more instruction or *orientation and familiarity* with the purpose and pace of the exercises. At first participants expected dynamic exercises of high intensity, as opposed to the slower, mindful, and spiritual nature of the yoga and Pilates movements. Participants also identified *app issues*, particularly with the calendar and badges. The calendar was not saving events that users created and did not have buttons that were visually identifiable. Participants also identified badges that were not successfully being rewarded for completion of a task (eg, reading an article). Nevertheless, participants reported that the app was *ready to be delivered to the public*, assuming the minor issues with the app were revised (calendar and bug problems). When asked whether the app was ready for production, participants were satisfied with the exercise videos and articles, but suggested minor improvements to app function, as noted by Participant 4:

I think it [the app] requires just a little bit of tweaks, but as far as the exercises and things that are uploaded on it, yes

Based upon this qualitative feedback, the project team determined that after the app issues identified by participants were revised, that the app and content be set into production within the app marketplace.

### Creation of Exercise Content

The creation of app content resulted from an initial development phase that included 4 stages and several revision iterations. Regarding the initial development phase, the TEAMS exercise intervention was first derived from a comprehensive therapeutic program used at a specialized MS clinic in Birmingham, AL, which aimed to improve overall physical function and wellness of patients with MS through yoga, Pilates, and dual tasking exercises.

#### Stage One

The first stage began with discussions among the stakeholders and research team to determine the general structure of the exercise intervention, which included the intervention duration, exercise components, session duration, and session frequency. This stage also incorporated feedback from the focus group, which recommended that the exercise videos be customized for different functional levels and led by or included people with disabilities.

#### Stage Two

During the second stage, the project therapist and the adapted exercise specialist worked together to determine the specific poses and exercise duration for the intervention components. This resulted in the first version of the intervention, which included 20 one-hour exercise sessions consisting of yoga, Pilates, and dual tasking exercises.

#### Stage Three

The exercise content was further modified based on discussions between the adapted exercise specialist, the project therapist, the stakeholders, and the clinical consultants. These discussions included suggestions for adaptations to the poses and exercises for participants with different functional capacities. Following these discussions, a second version of the intervention was developed to include 4 levels of exercise adaptations to enhance the likelihood of including a more diverse group of people with MS with varying levels of functional mobility. Exercise progression was established across a 12-week intervention.

#### Stage Four

Stakeholders and clinical consultants conducted internal pilot tests on the intensity, frequency, and duration of the exercise videos. At the end of this stage, session details such as specific duration and repetition for each exercise movement and pose were finalized.

#### Revision Iterations Phase

The purpose of this phase was to obtain minor feedback on strategies to assign the 4 levels of exercise adaptations to participants and intervention delivery format for video production. Over a period of 3 months, the intervention was iteratively presented to the stakeholders, the research team, and the study consultants for their suggestions on further adaptations. The adapted exercise specialist and the project therapist, which included finalizing dual-tasking exercise content for each level of exercise adaptation, video scripts and exercise equipment, made minor revisions. In addition, 2 modified levels of exercise adaptation for people with osteoporosis were added based on feedback provided by the project consultants. The intervention was then prepared for video development and production and incorporated into the usability tests of the TEAMS app.

#### Exercise Equipment

The original onsite therapeutic exercise program in the clinic included several pieces of exercise equipment, which the project team adapted into a home-based package. After consultation with the clinical consultant, the total equipment list included the following items: yoga mat, yoga blocks, resistance bands, half roll, racket ball, yoga straps, sliders, Pilates disk, and a swiss, physio, and bouncing ball. The equipment was organized into 4 different home packages by each functional level.

### Final Product

**Figure 3:**
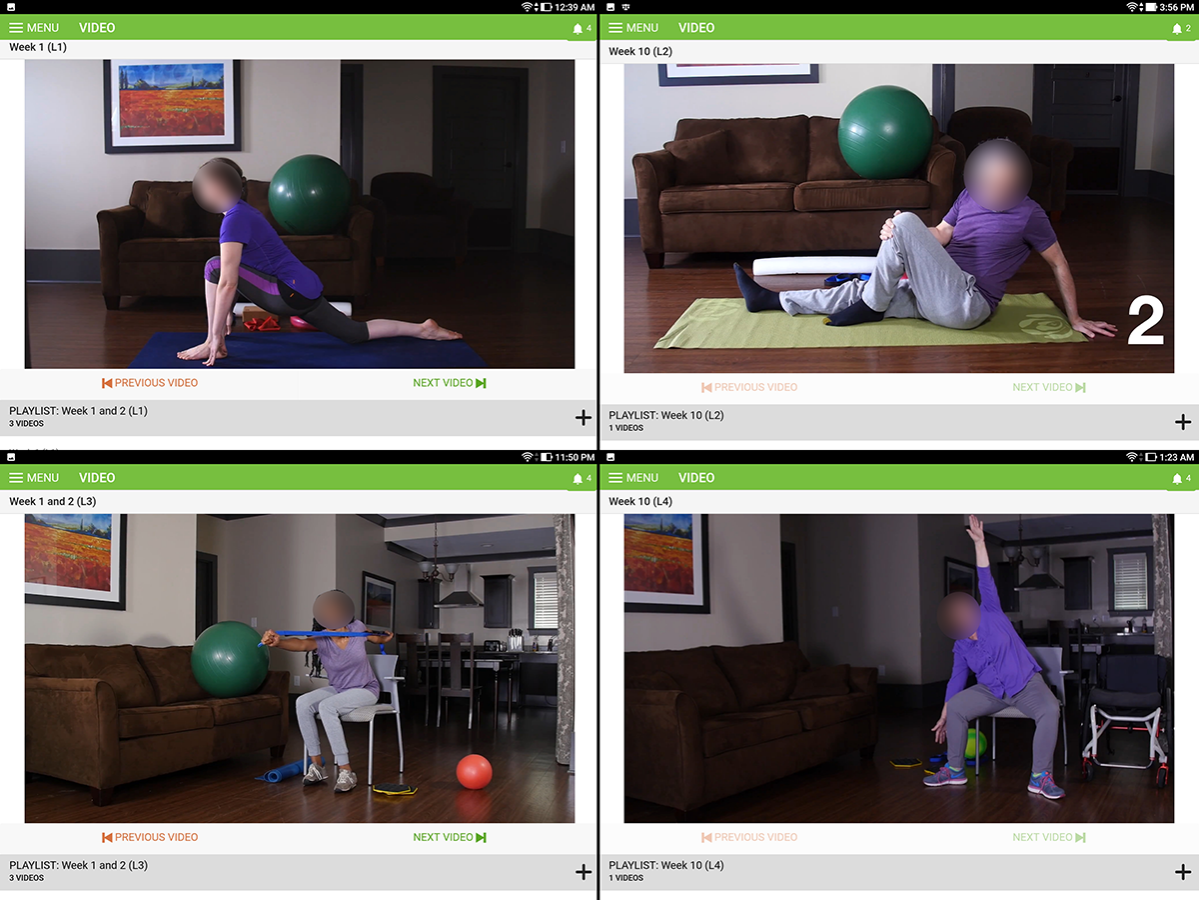
An example of exercises included at each of the four intervention levels (TEAMS 1-4).

After several revisions, the project team created an easy-to-use therapeutic exercise package that included a tablet, tablet stand, and a set of inexpensive exercise equipment to accommodate the videos. The resultant app included a password-protected feature that allows therapists to quickly alter the exercise videos to meet the functional needs of the individual through six different levels of TEAMS exercise adaptations (examples of the first four levels, TEAMS 1-4, shown in Figure 3):

- TEAMS 1: a level that included all yoga, Pilates, and dual-tasking exercises to be performed on the floor and in standing posture.
- TEAMS 2: a level that included all routines of yoga, Pilates, and dual-tasking exercises to be performed on the floor and in standing posture. All exercises were adapted for participants with MS with mild gait impairments.
- TEAMS 3: a level that includes all yoga, Pilates, and dual-tasking exercises being performed on the floor, in a chair in seated posture, and in standing posture. All exercises were adapted for participants with MS who experience more advanced gait impairments.
- TEAMS 4: a level that includes all yoga, Pilates and dual-tasking exercises being performed in a chair in seated posture. All exercises were adapted for people with MS who use a wheelchair as their main form of mobility.
- TEAMS 3OP: a modified version of TEAMS 3 for participants with MS who have osteoporosis. All exercises that involved trunk bending and twisting motions were removed,
- TEAMS 4OP: a modified version of TEAMS 4 for participants with MS who have osteoporosis. All exercises that involved trunk bending and twisting motions were removed.

**Figure 4:**
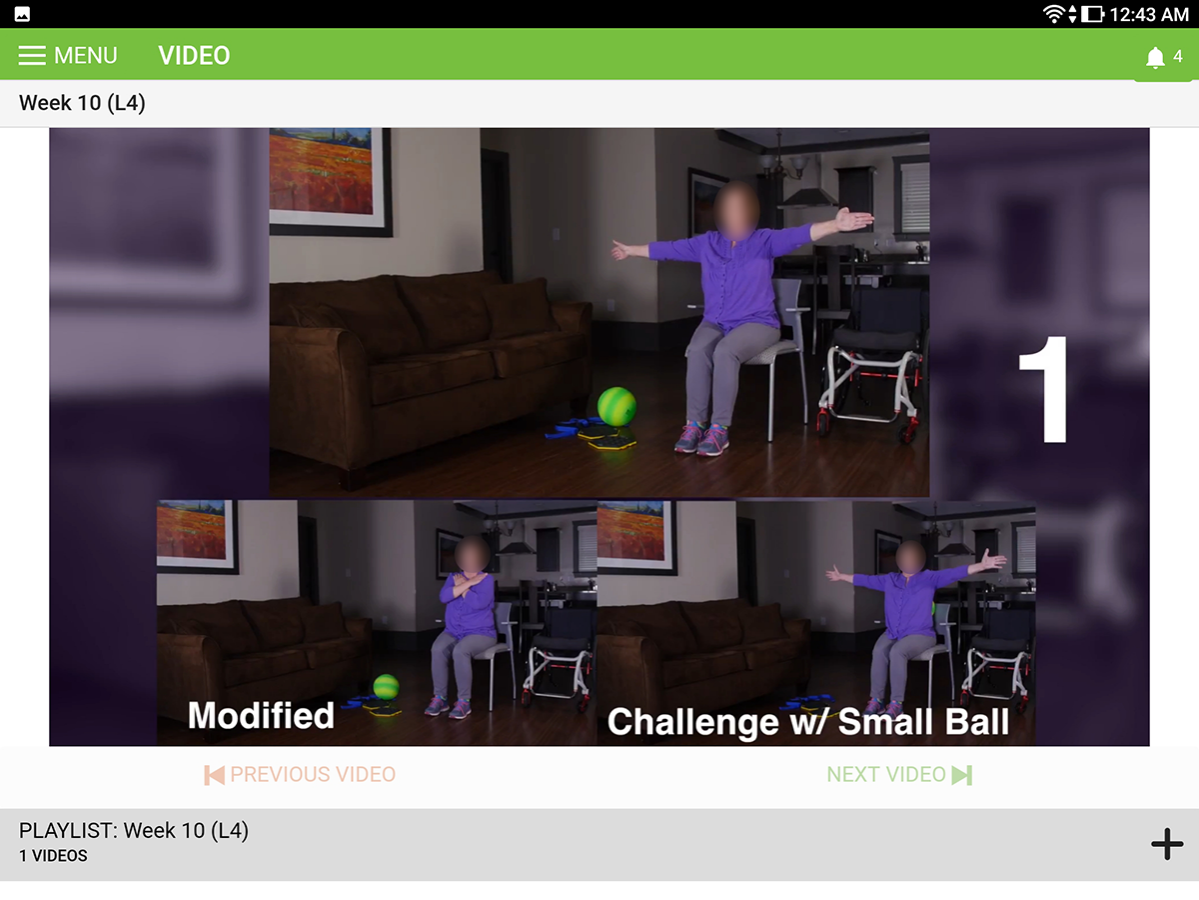
Example of movement modifications included in the app.

Within each level, most exercises also included modifications to increase or decrease the level of difficulty. Participants could choose to perform the standard exercises or modified version based upon their preferences (shown in Figure 4).

## Discussion

### Principal Findings

This paper describes the iterative development of an inclusive therapeutic exercise app for people with MS with self-reported Patient Determined Disease Steps scores from 0 to 7. The app is currently being used in an ongoing study targeting 820 adults with MS living in the Deep South (Alabama, Mississippi, and Tennessee). In accordance with the participant-centered focus of the agency that provided funding for this project (PCORI), nearly every stage of development was guided by extensive feedback from people with disabilities, including MS. A multidisciplinary team that included software developers, clinicians, consultants, and researchers complemented this feedback.

A novel component of this study was the heavy reliance on stakeholder feedback obtained through qualitative data from the mixed methods inquiry. These data informed the initial development phase, proof of concept testing, and final decision to terminate usability testing. Given that there are numerous ways of defining and measuring usability [31], the determination of an endpoint for usability testing is often hard to define. Within a mixed-methods approach, the research team utilized both quantitative and qualitative data to weigh the decision to halt usability testing. However, using this approach requires a few considerations. First, the research team or staff analyzing the mixed data should have a solid background in both qualitative and quantitative research and share the same ontological and epistemological beliefs. In this study the data analysts held theoretical assumptions within a meta-paradigm known as dialectical pluralism [34]. This approach emphasizes a mutual respect for both quantitative and qualitative data. Specifically, researchers should hold separate theoretical assumptions when analyzing both data types, before jointly merging the data for interpretation. Although this is not the only viable method of a mixed-methods investigation, it is important for the analysts to share similar belief systems. Otherwise, the analysts might disagree on their interpretations of the data or even the consideration of what in fact are *data*.

Second, a priori sample size determination is a highly debated issue within the extant literature for usability testing. Although Turner and colleagues have demonstrated that 5 participants are sufficient for the identification of usability issues [41], higher samples (eg, 10-12 participants) have been recommended [31]. Given that we assumed more problems would be identified in the early evaluations of the app, we chose to include 8 participants in the first usability test. With a more finalized app version, we incorporated a qualitative interview that used a qualitative sampling technique referred to as *saturation* (recruitment of participants until no new relevant themes emerge from their feedback) [42], to determine the final sample size. This method may be a useful tool to enhance the science of usability testing, as our resultant sample size of 5 did agree with usability recommendations [41]. However, since saturation requires analysts to interpret themes that are relevant to the usability tasks that participants perform, the nature of the usability tasks could influence the size of the sample required for saturation to be achieved. In other words, more complex tasks or products could require larger samples than simple tasks (as performed in the present study), but this notion requires further investigation.

### Limitations

This study had limitations. First, participants involved with the usability tests were active exercisers from a fitness facility with adapted exercise programs for people with disabilities. While this active population provides valuable insight towards issues that may prevent successful maintenance of exercise behavior, understanding perceptions of inactive people with MS may help identify issues during the adoption of exercise behavior. To minimize the impact of this limitation, the project team included 8 stakeholders who were inactive adults with MS though greater involvement of the target population would enhance the methodological rigor. Second, although the proof of concept testing included participants with MS, the early stages of development included individuals who had disabilities, but did not have MS. This was done to provide a convenient representation of different impairments (eg, poor vision, manual wheelchair use, power wheelchair use, and the use of other orthotic and walking devices). Third, although saturation was achieved for the qualitative data, the sample size was still small and may not adequately represent the heterogeneous participant characteristics that we might expect in the MS population. Fourth, this study did not contain objective criteria to indicate whether the exercises were suitable for people with MS. Fifth, this study did not incorporate assessments of app feasibility (ie, measures of use at the home) and did not base the exercises off of previously published studies, and, thus, will require evaluation of both the app and the effects of the exercises throughout an exercise intervention.

### Conclusions

The promotion of physical activity through mHealth exercise apps will require adaptation of the app and content to match the preferences and functional abilities of people with disabilities. Participants and stakeholders identified several exercise components that required further modification. Participant feedback also heavily impacted the development of app features and functions. Following several iterative evaluations, the project team and participants finalized an exercise app that can be easily operated in the convenience of the home and tailored to the functional needs of individuals with MS. The latest version of the TEAMS app is incorporated into an ongoing randomized controlled trial [43]. Collectively, the study findings emphasize the importance of user-centered designs that include participants throughout several stages of the development process and utilize both quantitative and qualitative data for usability evaluations.

## Appendix

Multimedia Appendix 1 Usability test 1: classification within the usability problem taxonomy.

## Abbreviations

mHealth: mobile health
MS: multiple sclerosis
PCORI: Patient-Centered Outcomes Research Institute
SCT: Social Cognitive Theory
TEAMS: Tele-Exercise and Multiple Sclerosis
UCD: user-centered design
UPT: Usability Problem Taxonomy
