# Supplementary material for: TEAMS (Tele-Exercise and Multiple Sclerosis), a Tailored Telerehabilitation mHealth App: Participant-Centered Development and Usability Study
Source: JMIR Mhealth Uhealth. 2018 May 24;6(5):e10181. doi: 10.2196/10181 (PMC5992455; doi:10.2196/10181)
Supplement: Multimedia Appendix 1 [file mhealth_v6i5e10181_app1.pdf]

Appendix 1. Usability test 1: classification within the Usability Problem Taxonomy				
Usability Problem Description	Place of Occurrence	UPT Classification Artifact	UPT Classification Task	Severity
User looked for a search screen with magnifying glass when trying for perform a task.	Main Menu Screen	Visualness-Object Screen Layout	Task Mapping-Functionality	1
Could not find definition for technical terms in articles	Article Screen	Language-On-screen text	N/C	3
Had difficulty reading print in article and could not enlarge screen	Article Screen	Visualness-Object Appearance	Task-Mapping-Functionality	3
No reminders/prompts to exercise when they have not performed required workouts	Main menu Screen	Language-Other Wording-Feedback messages	Task-Facilitation-Keeping the user task on track	1
No prompts telling the user "Good Job"	Video Screen	Language-Other Wording-Feedback Messages	Task-Facilitation-Keeping User on task on track	1
App lacking color	All screens	Visualness-Object Appearance	N/C	1
No backup plan provided if user unable to perform assigned workout	Calendar	N/C	Task-Mapping-Functionality	3
Not sure where to touch the screen to begin survey	Survey Screen	Manipulation-Visual Cues	Task-Mapping-Navigation	2
When you choose an answer in a survey the field turns the same color as the labels	Survey Screen	Visualness-Object Appearance	Task-Mapping-interaction	2
Not intuitive as to which menu to select to add a friend.	Main Menu	Manipulation-Visual Cues	Task-Mapping-Navigation	3
Difficult to figure out how to send an message to a friend	Main menu screen	Manipulation-Visual Cues	Task-Mapping-Navigation	3
Hit wrong button often	Main menu screen	Visualness-Object Layout	Task-mapping-Interaction	2
No confirmation that a survey was saved	Survey Screen	Language-Feedback Messages	Task-mapping-interaction	3
Backwards triangle at	Main Screen	Visualness-Object	N/C	2

bottom of screen was frequently pushed instead of back button on app		Appearance		
Very easy to remove a friend accidentally.	Users Screen	Manipulation-Cognitive Aspect	Task Facilitation-User Reversal Action	3
When you click on a friend in the newsfeed it opens your own profile and not the friends.	Newsfeed Screen	N/C	Task-Mapping-Navigation	3
The fact that there was no way to copy text made navigation difficult	Newsfeed	N/C	Task-Facilitation-Automation	2
Not apparent whether a post was successfully "liked"	Newsfeed	Language-Feedback Messages	Task-mapping-Functionality	2
When making a post, the green Post button is not viewable when typing. This creates confusion as to how to complete the process of creating a post.	Newsfeed	Visualness-Object Layout	Task Facilitation-Keeping the user task on track	3
No help tutorial to explain how to use the app if a user experiences problems	N/A	N/C	Task-mapping-Functionality	4
Cannot track progress on calendar or anywhere else in app	Calendar	N/C	Task-Mapping-Functionality	2
No explanation as to how app will benefit the user	N/A	N/C	Task-mapping-Functionality	1
No ability to input pain or level of wellness before or after workout	N/A	N/C	Task-mapping-functionality	2
Not intuitive how to create a new event in the calendar	Calendar	Cognitive Aspect-Visual Cues	Task-mapping-Navigation	2
Post not appearing on screen after being saved	Newsfeed	Visualness-Presentation of result	Task-mapping-interaction	3
